# Non-Invasive Diagnostics of Male Spermatogenesis from Seminal Plasma: Seminal Proteins

**DOI:** 10.3390/diagnostics13152468

**Published:** 2023-07-25

**Authors:** Michal Ješeta, Anna Pospíšilová, Lenka Mekiňová, Kateřina Franzová, Pavel Ventruba, Eva Lousová, Bartosz Kempisty, Tomáš Oždian, Jana Žáková, Igor Crha

**Affiliations:** 1 Center of Assisted Reproduction, Department of Gynecology and Obstetrics, Masaryk University Brno and University Hospital Brno, 62500 Brno, Czech Republic; mekinova.lenka@fnbrno.cz (L.M.); franzova.katerina@fnbrno.cz (K.F.); ventruba.pavel@fnbrno.cz (P.V.); lousova.eva@fnbrno.cz (E.L.); zakova.jana@fnbrno.cz (J.Ž.); crha.igor@fnbrno.cz (I.C.); 2 Department of Veterinary Sciences, Czech University of Life Sciences, 16500 Prague, Czech Republic; 3 Department of Animal Physiology & Immunology, Faculty of Science, Masaryk University, 60200 Brno, Czech Republic; 499418@mail.muni.cz; 4 Department of Veterinary Surgery, Institute of Veterinary Medicine, Nicolaus Copernicus University, 87-100 Torun, Poland; kempistybartosz@gmail.com; 5 Department of Human Morphology and Embryology, Division of Anatomy, Wrocław Medical University, 50-368 Wrocław, Poland; 6 Physiology Graduate Faculty, North Carolina State University, Raleigh, NC 27695, USA; 7 Laboratory of Experimental Medicine, Institute of Molecular and Translational Medicine, Faculty of Medicine and Dentistry, Palacký University, Hněvotínská 5, 77900 Olomouc, Czech Republic; ozdiant@seznam.cz; 8 Department of Health Sciences, Faculty of Medicine, Masaryk University, 62500 Brno, Czech Republic

**Keywords:** spermatozoa, azoospermia, sperm isolation, sperm analyses

## Abstract

The compounds of seminal plasma have great potential as biomarkers of male fertility and can be used as a diagnostic tool for types of azoospermia. Azoospermia occurs in approximately 1% of the male population, and for an effective therapy of this form of male infertility, it is important to distinguish between obstructive and non-obstructive azoospermia. Proteins in seminal plasma can serve as biomarkers for diagnosing azoospermia. Considering the various types of obstructions, a combination of multiple proteins is advisable for diagnostic purposes. In this context, testicular and epididymal proteins are particularly significant, as they are specific to these tissues and typically absent in ejaculate during most obstructions. A combination of multiple biomarkers is more effective than the analysis of a single protein. This group of markers contains *TEX101* and *ECM1* proteins, combined detections of these two bring a diagnostic output with a high sensitivity and specificity. Similar results were observed for combined detection of *TEX101* and *SPAG1*. The effective using of specific biomarkers from seminal plasma can significantly improve the existing approaches to diagnosis of the causes of male infertility.

## 1. Introduction

Azoospermia (no sperm in ejaculate), a severe form of male infertility, affects around 1% of men globally. It may be caused by many factors, including blockage of the reproductive tract, hormonal disbalances, ejaculation problems or issues with spermatogenic epithelium or testicular function. Treatment of azoospermia strongly depends on the cause. If live sperm are produced, they can be retrieved from the testes, epididymis or vas deferens for further IVF therapy (most often for the intracytoplasmic sperm injection method). Three types of azoospermia are described—pre-testicular, testicular and post-testicular.

(a) Pre-testicular azoospermia includes cases of congenital, acquired and idiopathic hypogonadotropic hypogonadism or secondary testicular failure. This is a state when only a small or zero amount of sex hormones is produced due to a disruption of the hypothalamic–pituitary axis. It is associated with poor nutrition, the use of medications (narcotics, chemotherapies), pituitary tumors, trauma or low testosterone (hypogonadism). Therapy of such conditions is possible using hormonal substitution.
(b) Testicular azoospermia is caused by disorders in the development of testicular tissue. The disorders can be congenital (undescended testicles, Klinefelter’s syndrome, Sertoli cell-only syndrome), caused by two genetic errors: chromosomal abnormalities or deletions in the Y chromosome. Acquired causes are mainly infections, gonadotoxic chemicals, cancer, testicular trauma or gonadotoxic therapy. It can also be associated with varicocele or a low level of testosterone.
(c) Post-testicular azoospermia caused by post-testicular abnormalities is usually commonly treatable. The most frequent cause of post-testicular azoospermia is an obstruction in the genital tract (obstructive azoospermia—OA). In patients with cystic fibrosis, CBAVD (congenital bilateral absence of vas deference) is present. The most frequent OA is epididymal obstruction (EO) caused by infection (chlamydia, trichomonas, mycoplasma, ureoplasma, adenovirus, etc.), iatrogenic, traumatic or idiopathic causes. This form of azoospermia can be caused by retrograde ejaculation brought on by surgical procedures or antipsychotic or antidepressants drugs.

Many causes of azoospermia are curable and fertility can be restored. Men with OA who undergo the semen collection procedure using some of the sperm isolation methods have a high chance of successful collection in more than 90% of cases. Men with non-obstructive azoospermia (NOA) are usually diagnosed with a serious damage to the spermatogenesis itself. In such cases, only very small or no areas of testicular tissue with functional spermatogenesis are preserved. From men with NOA, semen is usually collected by biopsy (sometimes repeated); however, it is successful only in about 30% of cases. The remaining 70% of men with NOA can be subjected to unnecessary repeated biopsies which are often ineffective and may lead to a temporary decrease in serum testosterone, to testicular atrophy, or hypogonadism [1].

For this reason, it is crucial to diagnose the type of azoospermia correctly. In general, elevated levels of FSH indicate disrupted spermatogenesis, while a normal level is associated to obstructive azoospermia. Nevertheless, as extensive screening clearly demonstrates, the levels of plasmatic FSH cannot be considered a reliable tool to diagnose the type of azoospermia [2]. Men with high levels of gonadotropins (FSH, LH) and bilaterally small testicles have NOA. However, men with normal values of gonadotropins can have both OA and NOA. Testicular biopsy, as a frequently used method of diagnosing spermatogenesis, has a number of disadvantages. It is more complicated and more demanding for patients than seminal plasma analyses. But the main drawback is that testicular biopsy results (based on random sampling of testicular tissue) are often inaccurate due to the great heterogeneity of spermatogenesis in the testes [3]. From this point of view, noninvasive evaluation of spermatogenesis on the bases of consumption of seminal plasma brings new interesting approach in therapy of male infertility.

The aim of this work is to create a comprehensive literature review mapping the possibilities of non-invasive diagnostics of spermatogenesis based on the analysis of seminal plasma proteins. Given the large amount of proteins in seminal plasma and the high enzymatic activity of seminal plasma, it is very important to select specific and stable biomarkers.

## 2. Obstructive and Non-Obstructive Azoospermia

The OA covers more than 40% of all the cases of azoospermia. It is a condition in which normal endocrine and exocrine functions are preserved and sperm cells are produced in testicles, but they are not present in the ejaculate. The absence of spermatozoa in the ejaculate is caused by obstruction in the genital tract, which can be congenital or acquired. Obstruction can occur on the level of the rete testis, in the efferent ducts, in the epididymis or vas deferens [4].

Congenital OA can appear due to bilateral agenesis of vas deferens, which often occurs in patients with cystic fibrosis as a result of CTFR gene mutation [5]. A correct function of the CTFR protein is essential for prenatal luminization of vas deferens. Mutation of the CTFR disrupts the function of the chloride channel that regulates the concentration of chlorides and water in the lumen of the exocrine glands. The CTFR gene is highly expressed in the cells of epididymis, vas deferens and seminal vesicles, and therefore its mutations affect maturation and transport of spermatozoa. The organs that originate from the Wolf’s duct then produce viscous mucus. This leads to obstruction and degeneration of the vas deferens as early as in fetal development.

Epididymides and seminal vesicles are often rudimentary or absent altogether. Degeneration of vas deferens occurs after the 12–18th week of pregnancy [6]. Causes of acquired OA can be infections, trauma, iatrogenic injury or idiopathic epididymal obstruction [7].

While some of these conditions are curable, in most cases, semen must be collected directly from testes or epididymis and subsequent fertilization of oocyte must be performed by intracytoplasmic sperm injection (ICSI).

Approximately 60% of azoospermia cases are NOA, which is defined as the impossibility of sperm production in testicular tissue. It is thus a more serious form of azoospermia. NOA is further categorized into three main subtypes, hypospermatogenesis, maturation arrest and Sertoli cell-only syndrome:

- Hypospermatogenesis is defined as a condition in which all developmental stages of germinal cells are present in testes up to late spermatids, but their production is decreased [8].
- Maturation arrest is a condition in which the activation of androgen receptor on Sertoli cells is disrupted and, subsequently, the development of spermatozoa is ceased on the level of spermatocyte, and thus maturation of the germinal cells is not completed [9,10].
- Sertoli cell-only syndrome describes a state when germinal cells are totally absent. Only Sertoli cells are present in the testicular tissue, filling the seminiferous tubules [11].

Most cases in which biopsy is not effective are represented by Sertoli cell-only syndrome and maturation arrest.

NOA is the reason for infertility in 10% of sterile men [12]. A genetic cause is found in 21–29% of them. The main genetic causes of this condition are chromosomal abnormalities (Klinefelter syndrome) and Y-microdeletions [13].

Klinefelter syndrome is characterized by the presence of an extra X chromosome, with karyotype 47, XXY, found in 80–90% of the patients with Klinefelter syndrome. In the remaining 10–20%, a higher-grade aneuploidy occurs, such as 48, XXXY, 49, XXXXY, 48, XXYY variants or 47, XXY/46, XY mosaic [14]. In patients with Klinefelter syndrome, germinal and Sertoli cells degenerate during puberty, together with hyperplasia of Leydig cells and fibrotization of the interstitium. It is rare that spermatogenesis can be preserved in the seminiferous tubules.

With regard to the high prevalence of genetic abnormalities in men with NOA, it is recommended to perform a screening of karyotype abnormalities and Y-microdeletions. Despite the progress in analysis of Genetic causes of NOA, there are still 50–80% of cases labelled as idiopathic azoospermia [15].

However, NOA can also be caused by external factors, such as varicocele with enlarged plexus of testicular veins, which disables proper blood flow and can lead to overheating and oxidative stress to germinal epithelium [16,17]. Other causes can be endocrine disorders, foreign substances, cryptorchidism, viral diseases (mumps, HPV), testicular failure or gonadotoxins [17,18].

## 3. Seminal Plasma

Seminal plasma is the liquid compound of ejaculate providing protection, nutrition and transport of sperm cells [19]. It is a complex fluid formed of secretions of several accessory glands creating about 95% of the final ejaculate volume. Testes with epididymides produce about 2–5% of the seminal plasma; 30% is produced in prostate, 65–75% is produced in seminal vesicles and 1% is produced in bulbourethral and periurethral glands [20]. Its composition can vary not only among individuals but also among ejaculates of one person [21].

Production of seminal plasma in multiple tissues enables employment of many physiological mechanisms covering, for example, transport, nutrition, capacitation of spermatozoa or interactions with tissues of the female genital tract [19]. Seminal plasma contains large amounts of carbohydrates, lipids, inorganic salts, antioxidants, hormones, cytokines, steroids, cellular DNA, RNA, miRNA, peptides and proteins [22]. It also contributes to the buffering capacity of the ejaculate, which is necessary for the prevention of fructolysis and subsequent immobilization of spermatozoa and eventual death of sperm cells due to low pH [23].

It was found that there is quite a large variability in the protein composition of the seminal plasma of fertile men [24]. Seminal plasma is influenced by a variety of factors that may impact its quantitative and qualitative biochemical composition (ejaculation abstinence, age or genetic factors). From farm animals, the impact of nutrition is well known. Analysis of seminal proteins is not associated with psychological impact on patients. Seminal plasma is the material obtained during procedure of the standard spermiogram evaluation. Effective analysis of these biomarkers would be positive for further therapy of an infertile couple.

Although seminal plasma contains a large number of compounds with a potentially diagnostic value, it is rarely used for these purposes. Nevertheless, with the development of analytic approaches and with the growing number of studies on the composition of seminal plasma, it is increasingly considered as a matrix suitable for diagnostics of male infertility [25]. Compared to testicular biopsy, seminal plasma analysis methods have a number of advantages: they are non-invasive and do not involve any other painful procedures beyond the standard spermiogram examination. Indeed, with the right choice of biomarkers, such analysis can provide a comprehensive picture of the state of spermatogenesis, which biopsies cannot guarantee due to the different level of spermatogenesis in different areas of testicles.

A typical example of this modern analytical approach could be proteomic analysis, which was used for the discovery of most of the potential protein biomarkers discussed in this review. Unfortunately, typical proteomic workflow is the answer to the question of why there are not many new biomarkers used in routine diagnostics yet.

We can provide the case of *TEX101* (testis-expressed 101) as an illustration of such approach. The first phase of biomarker discovery is called the discovery phase. It is usually low throughput and quite expensive, but provides the most complex results. In our example, there were about 2300 proteins identified in five controls and five post-vasectomy men [26]. In the following step, the most promising proteins were submitted to targeted mass spectrometry analysis, which is cheaper and allows higher throughput. In this case, 31 most promising proteins was analyzed in 30 individuals with normozoospermia, non-obstructive azoospermia and vasectomy [27]. This step allowed to filter the list of proteins from 31 to 3 best candidates (with *TEX101* among them) at higher amounts of patients. The study continued with narrowing the list of protein biomarker candidates and increasing the number of patients—in this case, 18 proteins from the previous study were analyzed in 119 individuals using targeted mass spectrometry. As a result, the combination of *TEX101* and *ECM1* showed an almost exclusive discrimination between the azoospermia conditions [28]. The next step in this case is the development of the ELISA kit since mass spectrometry is an expensive technique requiring skilled operators, whereas ELISA is a sensitive and reproducible method which could be performed at more laboratories with less equipment needed [29]. Then, the diagnostic kit was finished, just waiting for adoption by the scientific and medical community. This could be a more difficult task than the actual biomarker development, as in case of *TEX101*, there is only one reference actually using the *TEX101* kit [30]. The references of using the biomarker kit in the diagnostic practice are extremely important as they provide real-world experience with the biomarker in more laboratories and more patients than is possible to provide by the original authors.

The pipeline discussed in the previous paragraph addresses several issues—first, there is a need for identification of as many proteins as possible. This is not feasible to perform on a large amount of patients due to time and financial costs. However, the protein changes could be caused by multiple reasons, e.g., the inter-individual or time variability of seminal plasma [31]. Thus, in further steps, it is necessary to increase the number of patients tested. The only rational way to increase the number of patients is to use more cost-effective methods. The trade-off is that those methods allow to detect less protein species; thus, the rational filtering of biomarker candidates is necessary. At the end of the development, there should be a method which is simple to use and allows the diagnosis of a reasonable amount of patients at costs acceptable in a particular treatment regime.

## 4. Proteins of Seminal Plasma

Proteins form a basis of seminal plasma; they are found here in a concentration of 35–55 mg/mL [19]. In 2013, 699 proteins of seminal plasma were detected, of which 83 were identified as testicular proteins, 42 proteins were from epididymides, 7 proteins were from seminal vesicles and 17 proteins were from prostate [32]. Approximately 60% of all the proteins of seminal plasma contribute to its enzymatic activity. More than 8% of seminal plasma proteome is formed by hydrolases and peptidases, about 4% is the inhibitors of proteases [26,32,33].

From the diagnostical point of view, the most interesting proteins are those that can be used as biomarkers of the level of spermatogenesis. In azoospermic patients, mainly specific testicular proteins and specific epididymal proteins are important as potential biomarkers suitable for distinguishing obstructive and non-obstructive azoospermia. In case of obstructive azoospermia, secretions of testes and epididymides are usually not present in the ejaculate, and thus the absence of these specific proteins in azoospermic patients is a typical sign of obstructive azoospermia. In patients with this diagnosis, it is necessary to collect sperm cells directly from the epididymis or the testicular tissue. The combination of MESA (aspiration of fluid with sperm cells from epididymis) and TESE (surgical removal of testicular tissue and isolation of sperm cell directly from the tissue) techniques are used. If seminal plasma of an azoospermic patient contains high levels of specific testicular and epididymal proteins at the same time, then the probability of successful isolation of sperm cells directly from the testicular tissue is very low. A reliable method for distinguishing OA and NOA based on composition of seminal plasma could prevent useless realization of these traumatic surgical procedures.

### 4.1. Proteins of Testicular Origin

Many diagnostically interesting testis-specific proteins with a general presumptive biomarker function can be found in seminal plasma.

A variety of proteins produced in testicular tissue has been identified. They include, for example, L-lactate dehydrogenase C (*LDHC*), phosphoglycerate kinase 2 (*PGK2*) and transketolase-like protein 1 (*TKTL1*). These proteins occurred in normal concentrations in all control samples of seminal plasma from fertile men; however, they were absent not only in samples from men after vasectomy and in men with OA, but also in men with the NOA diagnosis. Production of these proteins is often directly proportional to the intensity of spermatogenesis. This is why they have the potential to be used as markers of a proper spermatogenesis, although they cannot serve as markers for distinguishing OA and NOA [32].

**Figure 1:**
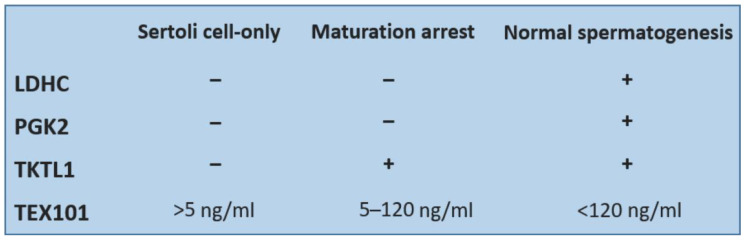
Comparison of detectability of four testicular proteins in seminal plasma of patients with Sertoli cell-only syndrome or maturation arrest and men with normal spermatogenesis.

- *LDHC* belongs to the family of lactate dehydrogenases which catalyze the conversion of pyruvate to lactate when reduced NAD+ is produced by the oxidation of NADH during anaerobic glycolysis. The presence of *LDHC* in testicular tissue was demonstrated in experiments on mice [34]. *LDHC* is expressed in both spermatocytes and spermatids. Immunohistochemical methods confirmed the presence of *LDHC* in the cytosol of spermatocytes and spermatids, while in spermatozoa it is located in the flagellum [35]. It is a testis-specific enzyme working as a catalysator of glycolysis with a significant effect on sperm motility and capacitation [36]. The lack of *LDHC* leads to the inhibition of glycolysis and results in a lower level of ATP which is necessary for the movement of the sperm flagellum. It is coded by a germ-cell-specific gene [37].
- *PGK2* is one of the key enzymes for the process of glycolysis, as it catalyzes the first step in the production of ATP. It is also a protein associated with fertility disorders, significantly affecting sperm motility and participating in the activation of phosphoglycerate kinase that is necessary for sperm development. Its expression takes place mainly in elongated spermatids [37,38]. *PGK2* is localized mainly in the region of sperm flagellum, where ATP is produced [39].
- *TKTL1* is a protein that serves as a catalyst for the conversion of seduheptulose-7-phosphate and D-glyceraldehyde-3-phosphate to D ribose-5-phosphate and D-xylulose-5-phosphate. This reaction connects the pentose cycle with the glycolytic pathway. Unlike for *LDHC* and *PGK2* that have the highest expression in spermatocyte and spermatid stages, the expression of *TKTL1* starts in spermatogonia. This fact could be used for distinguishing the subtypes of NOA. Men with good spermatogenesis have all three proteins, patients with maturation arrest have *TKTL1*; eventually, *TKTL1* and *LDHC* present in their seminal plasma. Men with Sertoli cell-only syndrome have none of these proteins present in their seminal plasma samples. In addition, *PGK2* and *LDHC* are important for sperm motility (Figure 1). These proteins could be used as biomarkers for prediction of a successful sperm retrieval [32].

A protein important for diagnostics is *TEX101* (testis-expressed 101). The gene for *TEX101* is located on the long arm of Chromosome 19 and is expressed only in the germinal cells in testes. It is not expressed in Sertoli and Leydig cells. It was demonstrated that *TEX101* is localized mainly on the plasmatic membrane of germinal cells throughout the gametogenesis, while in the cytoplasm, it is found only minimally [40]. The function of the *TEX101* protein is unknown, but it is presumed to be important during the sperm capacitation process and also play a role in sperm binding to zona pellucida [41]. The levels of *TEX101* above 120 ng/mL of seminal plasma were confirmed as a manifestation of normal spermatogenesis; values from 5 to 120 ng/mL are associated with hypospermatogenesis and values below 5 ng/mL indicate Sertoli cell-only syndrome.

More than 800 samples of seminal plasma from men with various diagnoses were used for detection of the *TEX101* protein with a detection limit of 0.9 ng/mL. This value enabled distinguishing men before and after vasectomy with a 100% sensitivity and a 100% specificity. Men after vasectomy, patients with Sertoli cell-only syndrome and men with obstructive azoospermia had only undetectable levels of the *TEX101* protein, while in seminal plasma of fertile men, the protein was well detectable [42].

Lipocalin-prostaglandin D synthase (L-PGDS) is a protein responsible for catalyzing the PGH2 precursor, which is essential for many other prostanoids. It is located mainly in Leydig cells, Sertoli cells and in the cells of excretory ducts of the testes and is produced into seminal plasma. The level of L-PGDS in seminal plasma is a good biomarker of the azoospermia type. In men with OA, the level of this protein was confirmed to be below 100 µg/L. Men with high levels of L-PGDS (above 100 µg/L) probably have NOA [43].

Serum amyloid P component (*SAP*) belongs to the group of pentraxin proteins with a less frequent pentameric structure. Found in blood serum, they are important for immunity as they recognize foreign antigens. *SAP* is also a protein of seminal plasma located on sperm flagellum and the tissues of testes and epididymides. Its concentration in seminal plasma correlates with spermiogram parameters [44].

**Figure 2:**
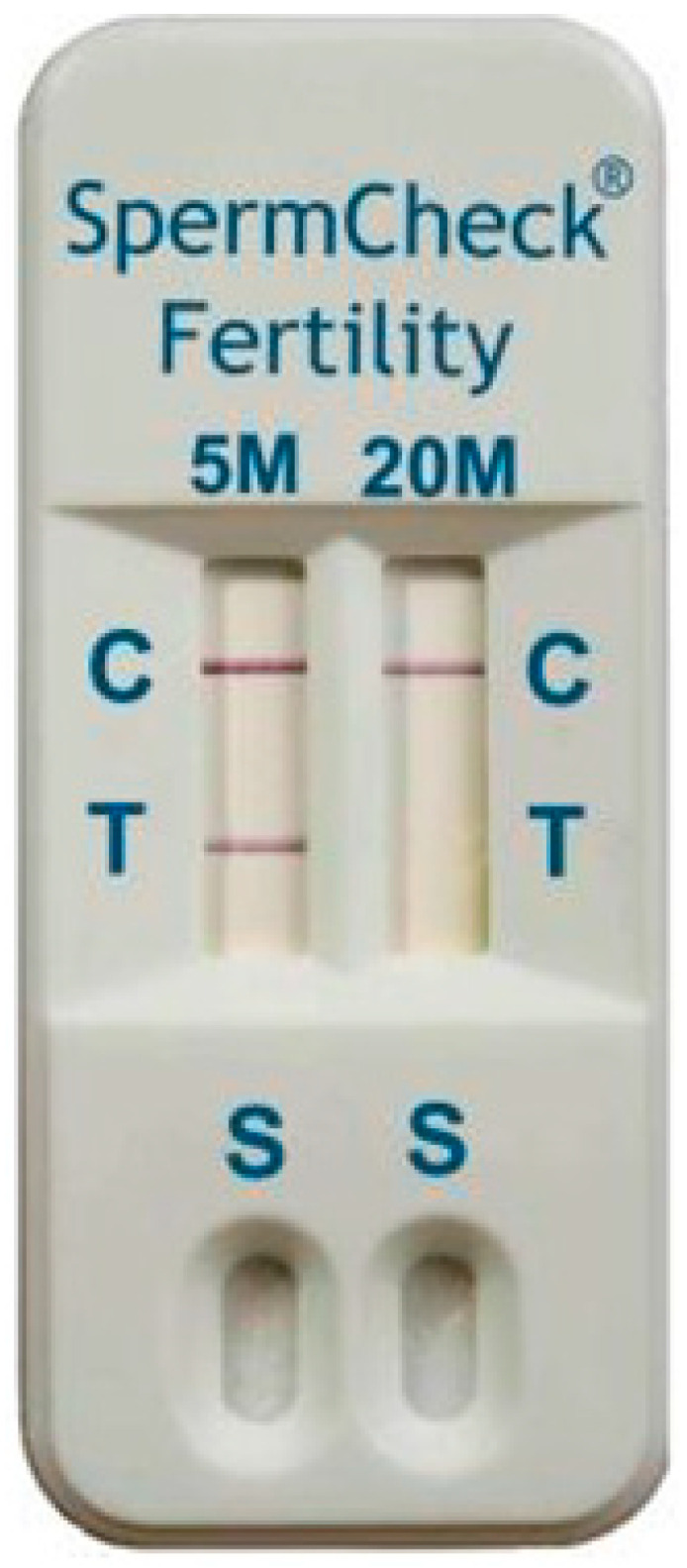
SpermCheck Fertility: Home immunodiagnostic test. The test is based on binding of monoclonal antibodies on epitopes of acrosomal antigen *ACRV1*. Diluted semen where *ACRV* is released into the buffer is applied to a well of the kit (S). There is colloidal gold conjugated with monoclonal anti-SP-10 antibodies on the absorbent pad in the cartridge. These antibodies are rehydrated, and they bind to the SP-10 antigen. The monoclonal antibodies specific for an independent epitope on the SP-10 protein bind to the complex of gold-antibody SP10 as they pass through the absorbent pad, which causes reddening of the line in the test area (T). Appearance of visible lines in the control area (C) of the test indicates that the device functioned properly and the fluid flowed through the test strips. This test shows the results of spermiogram with sperm concentration above 5 mil/mL, but below 20 mil/mL.

Acrosomal vesicle protein 1 (*ACRV1*) known as seminal protein 10 is expressed during the late phase of spermatogenesis [45]. In human tissues, *ACRV1* is found in round and elongated spermatids, mainly in the acrosomal membrane and the matrix of sperm cells and therefore it could have an effect on spermatogenesis [46]. The *ACRV1* protein was used for production of commercially available home tests suitable for distinguishing normozoospermia and oligozoospermia (SpermCheck Fertility^®^, DDC, London, UK; Figure 2) or azoospermia in men after vasectomy (SpermCheck Vasectomy^®^, DDC, London, UK). The test enables distinguishing normal, low and very low sperm concentration [47].

### 4.2. Proteins of Epididymides and Accessory Glands

Accessory sex glands produce the majority of the ejaculate volume. Seminal vesicles are most important in this respect. For distinguishing of OA and NOA, epididymal proteins are very important; they are produced independently on spermatogenesis. However, in the case of obstruction in the genital tract, they are usually not found in the seminal plasma. Their presence is strongly dependent on localization of the obstruction. If the obstruction is on the level of rete testis or the proximal part of epididymis, these proteins can be identified also in OA patients.

Glycoprotein *CRISP1* (cysteine-rich secretory protein 1), which is produced in epididymis, participates in sperm motility, capacitation and proper acrosomal reaction through its binding to the post-acrosomal region of sperm cells. In this way, *CRISP1* probably prevents premature sperm capacitation. A Western blot analysis confirmed that *CRISP1* is present in the seminal plasma of OA men in very low concentrations or is absent (depending on type of obstruction in the genital tract), and therefore it could be used as biomarker of OA/NOA in the future [48].

Niemann–Pick C2 (*NPC2*) is a protein that is not specific for epididymis. It is primarily produced in epididymis; however, in lower amounts, it is also produced in prostate, seminal vesicles and vas deferens. It has been demonstrated that its production decreases significantly in men after vasectomy, which suggests its theoretical potential to be used for distinguishing OA and NOA [49]. Samples from patients with azoospermia and samples from fertile men were analyzed by 2-D gel electrophoresis and *NPC2* was found in all the samples obtained from men with NOA while it was not present in the samples obtained from men with OA [24].

Glycoprotein *GGT7* (gamma-glutamyl transferase 7) is produced in several tissues of the male genital tract. The highest amounts are produced in seminal vesicles, lower in the epididymis, testes (all cells of germinal epithelium, Sertoli cells, Leydig cells) and prostate [50]. Very low expression of the *GGT7* protein was described in the seminal plasma of men with NOA [51]. *GGT7* is a strong antioxidant participating in the defence against reactive oxygen species [52].

Sorbitol dehydrogenase (SORD) is a protein involved in the mechanism of glycolysis. This enzyme increases the intensity of tyrosine phosphorylation and helps the conversion of sorbitol to fructose; in this way, it can participate in sperm capacitation. SORD is located directly on sperm cells and is expressed in the testicular tissue (Sertoli cells, Leydig cells), epididymis and prostate. SORD is not necessary for fertilization [53].

Extracellular matrix protein 1 (*ECM1*) is a protein released to ejaculate mainly from the epididymis. There are four variants of this protein. Seminal plasma contains the *ECM1a* variant. The *ECM1* protein can be used as a biomarker for distinguishing OA/NOA without testicular biopsy. Concentration of *ECM1* below 2.3 µg/mL of seminal plasma indicates obstructive azoospermia with a specificity of 73% and a sensitivity of 100% [28].

Prostaglandin synthase (PTGDS) is an enzyme produced by Sertoli and Leydig cells in testicles and also by epithelial cells of the prostate [26]. Among other functions, PTGDS contributes to the maintenance of the hemo-testicular barrier [54]. Its concentration in seminal plasma of men with fertility disorders is lower than in fertile men and it varies depending on the type of disorder [55]. A significantly lower concentration of PTGDS was found in samples obtained from men after vasectomy and with OA [43].

Sperm-associated antigen 11B (SPAG11B) is coded by the *spag11B* gene. This antimicrobial peptide is produced by epididymal tissue and also by the Leydig cells in testes. In large amounts, it is produced mainly by the epithelium in a proximal part of the epididymis [56]. It is important for sperm motility and it might have specific functions through association with specific receptors [57].

Another compound of seminal plasma proteome is the prolactin-induced protein (PIP) produced in testes, epididymides and seminal vesicles. It used to be described as a potential biomarker suitable for distinguishing OA/NOA [58]; however, later studies revealed that although PIP is not present in patients with OA, only about 60% of men with NOA have it in their seminal plasma. Therefore, it is not a specific biomarker and it is not suitable for distinguishing azoospermia types [24].

The main proteins of seminal plasma are semenogelin 1 and semenogelin 2 (*SEMG1*, *SEMG2*) produced in seminal vesicles, representing nearly 80% of the volume of total seminal plasma proteome [33]. These proteins ensure the typical viscous character of seminal plasma after ejaculation. *SEMG* is involved in the activation of hyaluronidase; it inhibits sperm motility and prevents its hyperactivation. It also suppresses the tyrosine phosphorylation important for capacitation [59]. Gradually, a proteolysis of semenogelins occurs, followed by liquefaction of the coagulum. The liquefaction is mediated by proteases such as PSA that is produced by the secretory cells of the prostate [21,60].

The ejaculate contains a range of other enzymatically active compounds, for example, nitrogen oxide synthases (NOS), transforming growth factor (TGFB) or protein C inhibitor (PCE) secreted in the seminal vesicles [33,61].

## 5. Proteins of Seminal Plasma as Biomarkers for Azoospermia-Type Diagnostics

During the comparison of men after vasectomy and men with normozoospermia, more than 2000 seminal plasma proteins were identified. Of these, several proteins expressed only in normozoospermic men were detected (*n* = 32). They were of testicular or epididymal origin, and they can be used as potential biomarkers for distinguishing OA and NOA [51].

This group contained the above-mentioned testicular proteins *LDHC*, *TEX101* and *PGK2* as well as other proteins of seminal plasma, such as the following:

- *DPEP3* (dipeptidase 3)—this glycoprotein belongs to the family of membrane-bound dipeptidases [62]. It is a protein specific for the testicular tissue, expressed in all cells of the germinal epithelium (spermatogonia, spermatocytes, spermatids, sperm cells). It creates a complex with the *TEX101* protein [63].
- ADAM7 (A Disintegrin and Metalloprotease 7) is an enzyme produced in epididymis and located on the surface of sperm cells. It probably plays a key role during sperm maturation in epididymis [64].
- *PGK2* (phosphoglycerate kinase 2) activates the phosphoglycerate kinase which is important for sperm development. It is a germ-cell specific protein [37].
- HIST1H2BA is a testis-specific nuclear histone that participates in chromatin condensation. Abnormal retention of chromatin in spermatozoa is a sign of immaturity. Therefore, this histone is sometimes referred to as a biomolecular marker of sperm quality. It has been documented that the expression of HIST1H2BA changed when the sperm cells were exposed to a higher oxidative stress or in the case of varicocele [65].
- *HSPA4L* (heat shock protein family a member 4Like) is expressed in testes in spermatogenic cells, mainly in the spermatocyte and spermatid stages [66].
- *SPACA3* (sperm acrosome membrane-associated protein 3) is a lysosomal protein expressed in spermatids, located on the acrosome of spermatozoa with a probable role of receptor for oocyte membrane saccharide N-acetyl glucosamine. Most likely, it is also involved in the sperm–oocyte interaction during fertilization [67].
- GAPDHS (glyceraldehyde 3-phosphate dehydrogenase) is coded by a germ-cell-specific gene expressed on spermatids [37]. It is a glycolytic enzyme located on the sperm flagellum, important for sperm motility [68].

For the serum amyloid P component (*SAP*) protein mentioned above, various representations in seminal plasma have been described in patients with various diagnoses. No function is attributed to the *SAP* protein influencing fertility; however, it is known to be present in the testicular tissue, namely in the peritubular zone of seminiferous ducts [69]. *SAP* was undetectable in samples obtained from men after vasectomy, while in the azoospermic men with Sertoli cell-only syndrome [70], its concentration in seminal plasma was in a strong positive correlation with sperm concentration; therefore, the protein could be used as a biomarker, for example, in cryopreserved samples or for the development of home fertility tests [44].

From the diagnostic point of view, proteins such as *NPC2* or the *CRISP1* glycoprotein are very interesting, both produced in epididymis and potential markers of azoospermia. For *CRISP1*, it would be advisable to supplement the analysis by determination of the activity of neutral alpha glucosidase (NAG) produced in epididymis since it is known that if an obstruction occurs on testicular or proximal epididymal level, *CRISP1* can still be detected. Spectrophotometric analysis of the NAG activity is a sensitive and non-invasive method suitable for localization of the obstruction in the male genital tract. Its activity positively correlates with the concentration of *CRISP* in the seminal plasma. With this approach, an 85% specificity and a 92% sensitivity were achieved for distinguishing obstructive and non-obstructive azoospermia [49].

Even more interesting than individual protein detection is the detection of protein combinations, for example, the combination of *TEX101* and the sperm-associated antigen 11B (*SPAG11B*). It was described that samples obtained from fertile men contained both *TEX101* and *SPAG11B* in normal concentrations. In men with OA, the level of both proteins was significantly decreased. On the contrary, in the samples obtained from men with NOA, the *SPAG11B* protein, produced by the Leydig cells, showed only a minimal decrease, while the concentration of *TEX101*, produced by the germinal epithelium, decreased rapidly [27].

**Figure 3:**
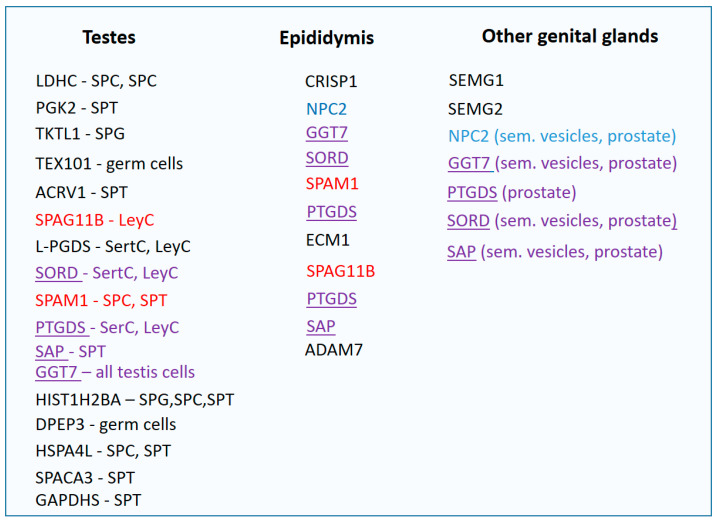
Proteins of seminal plasma divided according to the place of their expression. SPG—spermatogonium, SPC—spermatocyte, SPT—spermatid, SerC—Sertoli cells, LeyC—Leydig cells, red—proteins of testes and epididymides, blue—proteins of epididymides and other organs of genital tract, purple underlined—proteins produced in the entire genital tract.

The combined detection of *TEX101* and *SPAG1* yielded comparable results. The mere detection of *ECM1* led to the identification of 29 NOA samples (sensitivity of 73%, specificity of 100%). Subsequently, samples with *ECM1* concentration below 2.3 ng/mL were subjected to the analysis of the *TEX101* protein, and samples of five more men with NOA were added. The use of the combination of both proteins increased the sensitivity of NOA identification to 81%. At the same time, detection of *TEX101* below 0.9 ng/mL, OA and NOA could be distinguished with only a 32% sensitivity. Thus, *TEX101* itself is not a reliable biomarker of distinguishing OA and NOA [42]. Proteins suitable for the diagnosis of male fertility are shown in Figure 3.

## 6. Enzymatic Activity of Seminal Plasma

When interpreting the composition of seminal plasma, it is necessary to take into account its significant enzymatic activity: intensive proteolytic activity, dephosphorylation of phosphorylcholine or fructolysis. Seminal plasma contains large amounts of proteases and inhibitors of proteases. Proteases of the seminal plasma belong to several classes: metalloproteases, carboxypeptidases, aminopeptidases, and aspartate proteases, but most of them are trypsin or chymotrypsin serine proteases. Protease inhibitors are divided into cysteine, metalloproteinase, peptidase or serine inhibitors [33,71]. Seminal plasma proteins are continuously subject to proteolysis to form peptides, amino acids and nitrogenous compounds. Seminal plasma contains large amounts of proteolytic enzymes such as the prostate-specific antigen (PSA). PSA is a serine protease secreted by prostate and involved in the proteolysis of semenogelins, which leads to liquefaction of the ejaculate and subsequently to an increased sperm motility. There are various enzymes present in the seminal plasma, such as β-glucuronidase, β-galactosidase a β-N-acetylglucosaminidase. They bind to the head of a sperm cell during its passage through the epididymis [72]. Proteomic changes in the ejaculate seem to be associated mainly with metabolism and production of energy. From the metabolic processes, they are mostly linked to glycolysis, which is related to the activity of sperm cells in the semen [60].

The proteome of seminal plasma also contains glycosaminoglycan binding proteins responsible for the inhibition of premature sperm capacitation [73]. The proteins of seminal plasma maintain spermatozoa in inactive state up to the moment when liquefaction of the ejaculate enables their activation, allowing them to move and fertilize the egg [60].

Due to the high proteolytic activity, the stability of individual proteins of the seminal plasma may vary. To verify the stability of the tested proteins, samples of the ejaculate were first allowed to liquefy at room temperature for 1 h; then, they were centrifuged. After centrifugation, seminal plasma was separated from the cells and stored at −80 and at 4 °C. Seminal plasma was stored like this for 7 days, and concentration of *TEX101* in the samples was measured on a daily basis. At 4 °C, only a slight decrease in *TEX101* was recorded against the control. Therefore, the experiment revealed that even after 7 days of storage, there was no serious decrease in the *TEX101* protein concentration [29]. Similarly, it was described that storage of sperm samples at room temperature for the time of up to 6 h had no significant effect on the stability of the *ACRV1* protein and thus neither on the results of SpermCheck Fertility tests [47].

## 7. Conclusions

Parameters for determining the type of azoospermia are very limited and the only reliable method is biopsy. A great challenge for the future is the identification of reliable biomarkers that would enable avoiding the invasive procedures that can be very traumatic for the patient and can potentially lead to an irreversible damage to the testicular tissue.

The potential biomarkers are various types of molecules, such as free RNA, proteins or their combinations. At the moment, significant progress has been achieved in the field of proteomic analyses while searching for biomarkers of male sterility in seminal plasma and the proteins seem to be the most promising molecules for determination of the azoospermia type. For diagnostics, the key proteins are those from testes and epididymides. For testicular proteins, it is important that their production is independent of the spermatogenesis level. In the case of the epididymal proteins, the location of obstruction in the genital tract is crucial. Finding an optimal biomarker of this condition is not easy. From the practical point of view, detecting a combination of multiple proteins at once seems the most efficient.

Some tissue-specific proteins have already been used as biomarkers of male fertility. For detection of the type of azoospermia, proteins can be used individually (*L-PGDS*, *NPC2* or *ECM1*) or, more effectively, in a combination of two or more detected proteins with different expressions in the male genital tract. Testis-specific protein *TEX101* is used most frequently; it was also tested in combination with *ECM1*, specific for epididymis. Based on the different concentrations of these proteins, it is possible to distinguish OA and NOA with high specificity and sensitivity. Similar results were found for the combination of *TEX101* and *SPAG1* proteins. Moreover, the simultaneous detection of several seminal proteins (e.g., *TKTL1*, *LDHC*, *PGK2* and *TEX101*) can help to distinguish not only OA and NOA, but also the type of disorder and the level of spermatogenesis arrest.

Unfortunately, in patients high enzymatic activity and the associated high variability in seminal plasma protein composition was observed. For this reason, it is difficult to clearly define reliable specific biomarkers of spermatogenesis. Although the main biomarkers are defined, their effective use will require further research involving the active involvement of the clinical andrologist with a proteomic laboratory. However, the effective use of this approach can significantly improve the effectiveness of the existing approaches to diagnosing the causes of male fertility.

## Data Availability

Not applicable.
